# Construction and characterization of novel *Mycobacterium tuberculosis*-derived triple and quadruple knockout vaccines against tuberculosis

**DOI:** 10.1128/iai.00500-25

**Published:** 2026-04-21

**Authors:** Omar Garnica, Raja Veerapandian, Kishore Das, Abhishek Mishra, Varsha Rawat, Areanna Carmona, Vivek Chauhan, Rakesh Kumar, Jessica Chacon, Arshad Khan, Enrique I. Ramos, Shrikanth S. Gadad, Chinnaswamy Jagannath, Subramanian Dhandayuthapani

**Affiliations:** 1 Center of Emphasis in Infectious Diseases, Paul L. Foster School of Medicine, Texas Tech University Health Sciences Center El Paso 127363, El Paso, Texas, USA; 2 Department of Pathology and Genomic Medicine, Houston Methodist Research Institute & Weill Cornell Medical College 735417 , Houston, Texas, USA; 3 Department of Medical Education, Paul L. Foster School of Medicine, Texas Tech University Health Sciences Center El Paso 466553, El Paso, Texas, USA; 4 Department of Biological Sciences, University of Texas El Paso 171791, El Paso, Texas, USA; 5 Center of Emphasis in Cancer, Department of Molecular and Translational Medicine, Paul L. Foster School of Medicine, Texas Tech University Health Sciences Center El Paso 127365, El Paso, Texas, USA; Rutgers New Jersey Medical School Division of Infectious Diseases, Newark, New Jersey, USA

**Keywords:** tuberculosis, *Mycobacterium tuberculosis*, gene deletion, attenuated live vaccines, immunogenicity, protection, safety, SCID mouse

## Abstract

Tuberculosis (TB) is a deadly disease that claims the lives of over a million people each year worldwide. The Bacille Calmette-Guérin vaccine has long been used to protect against TB, but it produces variable effects across different populations and fails to protect against adult pulmonary TB. Therefore, there is an urgent need for alternative vaccines that can offer better protection. We have developed a strategy for the rational deletion of virulence-related genes in *Mycobacterium tuberculosis* (Mtb) to create hyperattenuation that also enhances immunogenicity. Previously, we generated both single (*∆fbpA*) and double knockout (DKO) (*∆fbpA-∆sapM*) mutants of Mtb and assessed their immunogenicity and efficacy using mice. Herein, we have created triple knockout (TKO) and quadruple knockout (QKO) strains to enhance the immunogenicity and safety of the DKO strain by deleting the *zmp1* and *dosR* genes. The resulting TKO strains, TKO-Z (*∆fbpA-∆sapM-∆zmp1*) and TKO-D (*∆fbpA-∆sapM-∆dosR*), and the QKO strain (*∆fbpA-∆sapM-∆zmp1-∆dosR*), were evaluated for their immunogenicity and safety in mice. Whereas TKO-Z and QKO strains exhibited superior immunogenicity compared to the DKO strain, their protective efficacy in mice was comparable. However, survival studies involving SCID mice indicated that the QKO strain was highly attenuated. Therefore, rational deletion of genes in Mtb seems to be an innovative approach for developing safer and more efficacious vaccines against TB.

## INTRODUCTION

Tuberculosis (TB), caused by *Mycobacterium tuberculosis* (Mtb), remains a primary cause of mortality worldwide, leading to approximately 1.6 million deaths annually (1). Though Bacille Calmette-Guérin (BCG) has been used as a vaccine against TB for nearly a century, it does not provide consistent protection against adult pulmonary TB (2). Moreover, its efficacy is limited against multidrug-resistant (MDR) and extensively drug-resistant (XDR) Mtb (3, 4). Control of TB is worsened by an increase in coinfections with both Mtb and HIV, because the latter depletes CD4 T cells (5). Thus, more efficacious vaccines against TB are urgently needed.

In this direction, various platforms based on subunit vaccines, recombinant BCG vaccines, and attenuated Mtb mutant vaccines have been developed (6–8). Some subunit vaccines that utilize Mtb immunodominant antigens, including the antigen 85 complex B (Ag85B), early secretory antigenic target-6 (ESAT-6), and culture filtrate protein 10 (CFP-10), have shown some promise as booster vaccines against TB (2, 9). Similarly, a few recombinant BCG strains that overexpress Ag85B have demonstrated significant protection against TB in preclinical studies (10–12). Furthermore, an attenuated Mtb mutant, MTBVAC, with deletions in the *phoP* and *fadD26* genes, is currently under clinical trials (13, 14). However, these advances have not resolved the need for an efficacious TB vaccine that surpasses the efficacy of BCG.

TB vaccine-induced immunity depends on the activation of Th1 cells and the release of immune molecules, such as interferon-gamma (IFN-γ), tumor necrosis factor-alpha (TNF-α), and interleukin-2 (IL-2), by CD4 and CD8 T cells (6, 15). The induction of such responses requires efficient antigen presentation by mycobacteria-infected antigen-presenting cells (APCs), which, in turn, depends on phagosome-lysosome (PL) fusion that precedes antigen processing and loading onto MHC-II molecules (16, 17). However, both BCG and Mtb have evasion mechanisms to avoid PL fusion that may affect the efficacy of current live TB vaccines and BCG (18, 19), which limits antigen presentation by APCs.

To overcome this barrier, we developed an Mtb-derived vaccine *∆fbpA*, which showed improved antigen processing through PL fusion (20, 21). The *fbpA* gene encodes mycolyltransferase, associated with mycolic acid synthesis in mycobacteria (22). It is also known for its fibronectin-binding activity and as a significant antigen (Ag85A) of the Ag85 family (23, 24). The *∆fbpA* strain showed protective efficacy against TB in mice similar to that of BCG (20). To improve efficacy, we deleted the *sapM* gene in ∆*fbpA* to result in a double knockout (DKO) vaccine (*∆fbpA-∆sapM)* (25). The product of the *sapM* gene, the SapM protein, is an acid phosphatase (26) that blocks PL fusion in the host cells by hydrolyzing phosphatidylinositol 3-phosphate (PI3P), a lipid critical for phagosome maturation and the recruitment of lysosomal machinery (27). The deletion of *sapM* in Mtb also prevents the hydrolysis of PI3P in infected macrophages, promoting PL fusion and facilitating improved antigen presentation (27). Consequently, the DKO strain not only demonstrated higher immunogenicity but also provided enhanced protection against TB, increasing memory T cells in mice compared to BCG (28).

Our data using the DKO strain led us to further improve this strain for higher immunogenicity and lower virulence. Herein, we have deleted two additional genes in the DKO strain. The gene *zmp1*, which encodes the Zmp1 metalloprotease (29), was selected because, previously, the deletion of *zmp1* in Mtb was found to increase PL fusion and inflammasome activation in macrophages (29). Others found that the deletion of *zmp1* in the genome of BCG enhanced its efficacy over wild-type BCG (30). We reasoned that deletion of *zmp1* would promote inflammasome activation and PL fusion, leading to improved antigen presentation and strong Th1 pathway activation. The second gene we chose for deletion was *dosR*, which encodes the transcriptional regulator DosR that controls the expression of over 50 genes in response to hypoxia related to dormancy and persistence in Mtb (31, 32). The *dosR* mutant Mtb strain has previously been reported to have lower virulence (33) and to modulate adaptive immunity in the host cells (34). We hypothesized that removing *dosR* would disrupt dormancy-related persistence and promote bacterial clearance, thereby enhancing vaccine safety.

By deleting *zmp1* and *dosR* in the DKO, we generated two triple knockout (TKO) strains known as TKO-Z (*∆fbpA-∆sapM-∆zmp1*) and TKO-D (*∆fbpA-∆sapM-∆dosR*), and a quadruple knockout QKO (*∆fbpA-∆sapM-∆zmp1-∆dosR*). Assessment of the immunogenicity and efficacy of the TKO and QKO strains in mice revealed that the TKO-Z and QKO strains are highly immunogenic, although their vaccine efficacy was only comparable to that of the DKO strain. However, we found that the QKO strain was highly attenuated in SCID mice, which exhibited survival rates closer to those of BCG-infected mice. Overall, our findings suggest that rational deletion of genes in Mtb may lead to the development of novel hyper-attenuated but immunogenic Mtb-derived vaccines against TB.

## MATERIALS AND METHODS

### Animals

Four- to six-week-old C57BL/6 mice were obtained from Jackson Laboratories (Bar Harbor, ME), and they were housed at the Laboratory Animal Resource Center at the Texas Tech University Health Sciences Center, El Paso (TTUHSC EP) or the Houston Methodist Research Institute (HMRI).

### Bacterial strains and culture conditions

Unless stated otherwise, all strains were grown at 37°C. *Escherichia coli* DH5-α (Invitrogen) was grown in LB broth/agar plates supplemented with antibiotics (ampicillin, 100 μg/mL; kanamycin, 25 μg/mL; or hygromycin, 100 μg/mL). *M. smegmatis* mc²155 and BCG were grown in 7H9/7H10 media with 0.2% glycerol, 0.05% Tween 80, and 10% ADC. *M. tuberculosis* strains were cultured in 7H9/7H10 with 10% OADC. Mtb mutant strains carrying antibiotic resistance cassettes were grown in 7H9/7H10 supplemented with kanamycin (25  μg/mL), hygromycin (50 μg/mL), or both.

*M. smegmatis* mc²155 (ATCC#70084), BCG (ATCC#35374), *M. tuberculosis* H37Rv (ATCC#27294), and *M. tuberculosis* Erdman (ATCC#35801) were obtained from ATCC. The *M. tuberculosis* ∆*fbpA*-∆*sapM* double knockout (DKO) strain, derived from Mtb H37Rv and used in this study, was previously reported (25).

### Construction of TKO-D, TKO-Z, and QKO mutant strains of *Mycobacterium tuberculosis*

Genomic DNA from Mtb strains was extracted using the CTAB method (35), while plasmids from *E. coli* were isolated using the QIAprep Spin Kit (Qiagen). DNA sequences for all genes and adjacent regions were downloaded from the NCBI database, and primers (Table 1) were custom-synthesized by Integrated DNA Technologies (IDT). The triple knockouts, TKO-D (∆*fbpA*-∆*sapM*-∆*dosR*) and TKO-Z (∆*fbpA*-∆*sapM*-∆*zmp1*) strains, were generated using the DKO strain (∆*fbpA*-∆*sapM*) as the base, while the QKO (∆*fbpA*-∆*sapM*-∆*dosR*-∆*zmp1*) strain was created using the TKO-D (∆*fbpA*-∆*sapM*-∆*dosR*) strain as the base.

**TABLE 1:** Oligonucleotide primers used in this study

Primers	Primer sequence (5′–3′)
DOSR1	GGACGGCCGCTGGTTCGGCAC
DOSR2	GCGCATATGGCACCACCTCGTGGTCATCG
DOSR3	ACGCATATGAAGCGCTCGCGGCCACCC
DOSR4	CCCGCTACGGTACCTCTGGCC
DOSR5	ACCCCGAGGTGCGGGTGGATCGGGCCATCG
DOSR6	CGGTCCTGCACCTCGACCAGCAGCTCGTGC
ZMPSE	CGGAACTAGTGACGGTTACCGAATCACGACGC
ZMPHIND	AGCGAAGCTTCACCCCGCCATCCTTCCACCTCT
ZMPXBA	AGCGTCTAGACAGGCCTTCGACGTCACCGAG
ZMPKPN	CACGGGTACCCGGCATTCCAGGCGCTGGAA
ZMP5	CGCAACTTCTCCACCACCTGCCGGTAGGG
ZMP6	CTCAACTGCCGATCGTGCTGTCGGTGGG
RV3310EX1	CATGAGGATCCCATGCTCCGCGGAATCCAGGCT
RV3310EX2	CGAGGATCCCTAGTCGCCCCAAATATCGGTTATTGG

To delete the *dosR* (*Rv3133c*) gene in DKO, we used the plasmid pKNOCK3133, which was constructed as follows. First, the 1 kb upstream and 1.2 kb downstream flanking regions of *dosR* were PCR-amplified using specific primers DOSR1 and DOSR2 for the upstream region and DOSR3 and DOSR4 for the downstream region, and cloned into the pCR2.1 vector separately. The DNA fragments comprising the upstream and downstream regions were excised from pCR2.1 and cloned into the p2NIL plasmid sequentially to create a single continuous fragment, resulting in plasmid pTBDOSR4. The cloned DNA fragment in this plasmid lacks not only the *dosR* gene region but also any antibiotic resistance gene. Following this, a DNA fragment containing *sacB*, *lacZ*, and *hgr* (hygromycin resistance) genes from pGOAL19 (36) was cloned into pTBDOSR4 at the PacI site to generate the knockout plasmid pKNOCK3133. This plasmid was electroporated into the DKO strain, and a markerless deletion of *dosR* was obtained using the two-step selection method of Parish and Stoker (36). The resulting mutant strain was named TKO-D.

To delete the *zmp1* (*Rv0198c*) *gene* in the DKO strain, we employed a phage transduction method instead. Briefly, we used PCR to amplify the upstream and downstream flanking regions of *zmp1* using the primers ZMPSPE and ZMPHIND, and ZMPXBA and ZMPKPN, respectively. These fragments were cloned into the 5′ and 3′ flanking regions of the *hyg* gene in the plasmid pJSC407 (37) to result in pMTB198. Subsequently, this plasmid was digested with PacI restriction enzyme and ligated with a similarly cut phasmid pHAE159 to result in the new phasmid pKNOCK0198. This was packaged using Agilent’s Gigapack III XL system and electroporated into *M. smegmatis* to produce mycobacteriophages. The temperature-sensitive mycobacteriophages were transduced (38) into the DKO strain, and the colonies were selected in 7H10/Hg plates. Colonies resistant to hygromycin were screened for the replacement of *zmp1* with the *hyg* gene by PCR. A few of these colonies were transformed with the plasmid pYO11 to remove the *hyg* marker. Finally, colonies sensitive to hygromycin, representing TKO-Z, were selected and confirmed by PCR. A similar approach was used to generate the QKO (∆*fbpA*-∆*sapM*-∆*dosR*-∆*zmp1*) strain from the TKO-D strain (∆*fbpA*-∆*sapM*-∆*dosR*).

### Confirmation of Mtb mutants

To confirm the deletion of the *zmp1* gene in TKO-Z strains and the *dosR* gene in TKO-D and QKO strains, we performed PCR using Taq DNA polymerase (Perkin Elmer, Foster City, CA) following standard protocols. Briefly, genomic DNA was extracted from each strain and used as a template. PCRs were set up in a 20 μL volume containing Thermo Scientific PCR Master Mix (2×) and ~100  ng of genomic DNA. Primer pairs DOSR5/DOSR6, ZMP5/ZMP6, and Rv3310EX1/Rv3310EX2 were used to amplify *dosR*, *zmp1*, and *sapM* loci, respectively. PCR products were analyzed on 1% agarose gels stained with ethidium bromide for visualization. Additionally, the gene deletions were verified by Southern blot and genome sequencing.

### Isolation of bone marrow-derived macrophages

Bone marrow-derived macrophages (BMDMs) were isolated as previously described by us (25, 39). Briefly, C57BL/6 mice were euthanized, and femurs were excised and cleaned of skin and muscle under aseptic conditions. Bone marrow containing hematopoietic stem cells was flushed from the bones using 5 mL of DMEM culture medium delivered through a syringe. The resulting cell suspensions were centrifuged, and cell numbers were determined. Cells were differentiated into macrophages by culturing in DMEM supplemented with 10% FBS and 10 ng/mL M-CSF at 37°C in 5% CO2 in Petri dishes.

### Intracellular survival assay

BMDMs (1 × 10^5^ cells) were plated in a 24-well plate and induced with recombinant mouse INF-γ for 12 h at 37°C in 5% CO_2_. H37Rv and its mutant strains were harvested from log phase cultures and used to infect BMDMs at an MOI of 1:10 for 4 h at a 37°C humid chamber with 5% CO_2_ in an antibiotic-free cell culture medium. Afterward, the cells were washed three times with sterile warm PBS and cultured in RPMI media, incubating for different time points. The cells were lysed with PBS containing 0.05% SDS, appropriately diluted, and plated onto 7H10 or 7H11 agar plates in triplicate and incubated for about 3 weeks at 37°C. Colonies were counted from three independent experiments.

### Immunofluorescence colocalization for phagolysosomal and autophagic markers

BMDMs (1×10^5^) were grown on a cover slip in a six-well tissue culture plate and infected with RFP-expressing Mtb H37Rv or mutant strains (DKO, TKO-D, TKO-Z, and QKO strains) as described previously (11, 25). Bacterial cultures were briefly sonicated to disperse the bacteria before infecting the BMDMs at a 1:1 MOI for 4 h at 37°C in 5% CO_2_. The cells were washed and further incubated for 48 h and 72 h with fresh cell culture medium. Cells were washed, fixed, and permeabilized as described previously. They were then stained overnight with anti-Rab7 and anti-LC3 antibodies, and the signal was visualized using FITC-conjugated anti-rabbit IgG. Colocalization was examined from 40 different fields from each slide and scored using a Nikon Ti fluorescence microscope equipped with a Metaview deconvolution software.

### Apoptosis assay

The apoptosis assay was performed as described earlier (40). Briefly, BMDMs (1 × 10^6^) were infected with BCG, H37Rv, and Mtb mutant strains (DKO, TKO-Z, TKO-D, and QKO) for 4 h with MOI 1:10. Following infection, cells were washed with warm sterile PBS and incubated for an additional 18 h at 37°C in 5% CO_2_. Apoptosis was assessed using the FITC Annexin V Apoptosis Detection Kit (BD Pharmingen), following the manufacturer’s protocol. Samples were acquired on a BD FACS Canto II flow cytometer (BD Biosciences, San Jose, CA), capturing 100,000 events per sample. Experiments were performed in triplicate for each strain, and apoptotic cells were analyzed using FlowJo v10 for Windows.

### *In vitro* antigen presentation assay

For determining the processing and presentation of Mtb antigens by Mtb-infected cells *in vitro*, BMDMs (1 × 10^5^ cells) were plated in a 24-well plate and induced with recombinant mouse INF-γ for 12 h at 37°C in 5% CO_2_. The monolayers were later washed two times with sterile PBS and infected with BCG, Mtb H37Rv, DKO, TKO-Z, TKO-D, and QKO at MOI of 1:5 for 4 h. Afterward, the monolayers were washed three times with sterile PBS and then overlaid with a 1:10 ratio of BB7-T cell hybridoma (1 × 10^6^) specific for Ag85B epitope (aa241–256), which was a kind gift from Dr. Clifford Harding and Dr. Henry Boom, Case Western Reserve University, OH. After 18 h, the cell-free supernatants were collected and tested for IL-2 secretion using an ELISA kit (BD OptEIA, BD Biosciences, San Diego, CA, USA) according to the manufacturer’s protocols.

### Vaccination of mice and isolation of the spleen

Six-week-old C57BL/6 mice, purchased from the Jackson Laboratory, were divided into six groups (*n* = 6). Mice in the naïve control group received 100 µL sterile PBS via the subcutaneous route, while the BCG control group and Mtb H37Rv and the mutant strains (DKO, TKO-Z, TKO-D, and QKO) group received 1 × 10^6^ bacilli subcutaneously once. At the end of the experiments (30 days after the first immunization), spleens were aseptically removed from the humanely euthanized mice and placed in 5 mL of RPMI medium. The spleens were then crushed with the plunger of a 10-mL syringe, passed through a 100 µM sterile cell strainer (Fisher Scientific), and finally centrifuged for 5 min at 300 × *g*. After centrifugation, the cell pellets were resuspended in 5 mL of ACK lysis buffer (Gibco Life Technologies) and incubated at room temperature for 5 min. Subsequently, 25 mL of sterile PBS was added, and the mixture was centrifuged at 300 × *g* for 5 min. The pellets were resuspended in 5–10 mL of RPMI medium containing 10% FBS, 100 I.U./mL penicillin, and 100 µg/mL streptomycin, and viable splenocytes were counted by Trypan blue staining using a BIORAD TC 20 automated cell counter.

### *Ex vivo* cytokine analysis

Splenocytes (2.5 × 10^5^) from naïve mice and mice immunized with BCG, Mtb H37Rv, and mutant strains were plated in a 96-well culture plate and induced with 20 µg of irradiated whole-cell Mtb lysate for 48 h at 37°C in 5% CO_2_. The culture supernatants were appropriately diluted and used to measure the concentrations of INF-γ, IL-1β, TNF-α, IL-2, and IL-12 using an ELISA kit (BD OptEIA, BD Biosciences, San Diego, CA, USA) following the manufacturer’s protocols. Plates were read at 450 nm using a Flexstation 3 plate reader (Molecular Devices).

### ELISPOT assay

We used ELISPOT to measure the frequency of IFN-γ producing cells in naïve or immunized mice with BCG or Mtb strains. Mouse IFN-γ ELISpot^Plus^ plates (Catalog #3321-4APT-10, MABTECH Inc., Cincinnati, OH) were washed three times with sterile PBS and then blocked with culture media (RMPI with 10% FBS). After washing with sterile PBS, splenocytes (2.5 x 10^5^ cells/well) from each mouse were seeded in ELISPOT plates and incubated with either 100 ng of PMA (for positive control), or 5 µg each cocktail of Ag85B and CFP-10 (BEI/ATCC), or culture medium (negative control) at 37°C in 5% CO_2_ for 48 h. Spots were developed using reagents supplied with the kit and following the manufacturer’s protocols. Plates were read using an *i*Spot ELISpot plate reader (Advance Imaging Devices GmbH, Straßberg, Germany). Spots from the negative wells were used to normalize the readings of the treated wells.

### Protective efficacy of vaccine strains after challenge

Six-week-old C57BL/6 mice (*n* = 6 per group) were immunized subcutaneously with BCG or Mtb strains (DKO, TKO-Z, and QKO), as previously stated, with PBS containing 1 × 10^6^ bacilli. Mice in the naïve control group received 100 µL sterile PBS. After 30 days of post-immunization, mice were challenged with 100 CFUs of Mtb (Erdman) bacteria/mouse through a GlasCol inhalation chamber, as reported previously (12, 28). The mice were humanely sacrificed after 30 days post-challenge, and lungs and spleen were extracted, homogenized in sterile PBS containing 0.05% Tween-80, and serially diluted. The dilutions were then plated onto Middlebrook 7H10 or 7H11 agar plates supplemented with OADC and incubated at 37°C for 3–4 weeks. After incubation, CFUs were enumerated to assess the bacterial burden in each organ. The reduction in CFU counts in vaccinated groups compared to the PBS control group was used as a measure of protective efficacy.

### SCID mice survival study

Eight- to nine-week-old SCID mice (B6.CB17-PrkdcSCIDSzJ/001913, Jackson Laboratory) (*n* = 6 per group) were maintained under specific pathogen-free conditions in individually ventilated cages. Animals were infected intravenously through the tail vein with 10^6^ CFUs (100 µL) of either BCG or Mtb wild-type or mutant strains (H37Rv, DKO, TKO-Z, TKO-D, and QKO). The control group of mice was injected with an equivalent volume of sterile PBS alone. Mice were monitored frequently for signs of distress. The humane endpoints for infected mice were set at 20% weight loss. Once the infected mice reached this condition, they were euthanized, and the days of survival were recorded to determine the survival curves.

### Statistical analysis

All experiments were performed in triplicate (unless otherwise stated), and data are presented as mean ± SD. Statistical analysis in this study was performed by using Student’s t-test with GraphPad Prism version 10.0 (GraphPad, La Jolla, CA). Survival curves were calculated by using the Mantel-Cox log-rank test (GraphPad, La Jolla, CA). Multiple groups versus control data in the efficacy studies were compared by one-way ANOVA with Dunnett’s post-test. *P*-values <0.05 were considered statistically significant.

## RESULTS

### Construction and confirmation of *M. tuberculosis* TKO-D, TKO-Z, and QKO mutant strains

**Fig 1:**
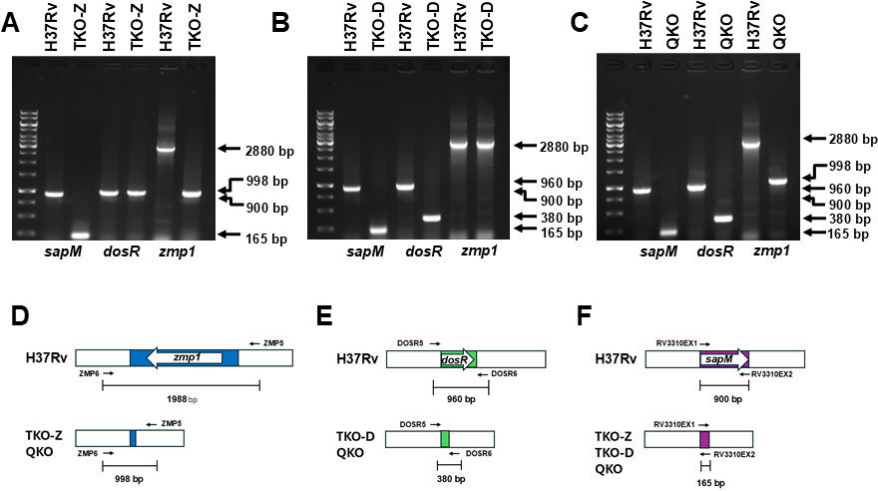
PCR confirmation for the rational deletion of genes in TKO-Z, TKO-D, and QKO strains. To confirm the deletion of *sapM*, *dosR,* and *zmp1* genes in TKO-Z, TKO-D, and QKO strains, PCR was conducted using gene-specific primers (primers Rv3310EX1 and Rv3310EX2 for *sapM* gene; primers DOSR5 and DOSR6 for *dosR* gene; primers ZMP5 and ZMP6 for *zmp1* gene) and genomic DNA templates from all three mutated strains and from the wild-type H37Rv. (**A–C**) PCR results for the *sapM*, *dosR,* and *zmp1* genes of the TKO-Z (**A**), TKO-D (**B**), and QKO (**C**) strains compared to wild-type H37Rv. The sizes of the PCR fragments obtained are shown on the right in base pairs (bp). The PCR results revealed appropriate gene deletion by amplifying smaller DNA fragments in the mutants than in the wild-type H37Rv, confirming the deletions. Diagrams in (**D**), (**E**), and (**F**) show the location of the primers around the *zmp1*, *dosR*, and *sapM* genes and the expected sizes of the PCR products from the wild-type H37Rv and TKO-Z, TKO-D, and QKO strains.

We have previously described the generation of DKO (*∆fbpA-∆sapM*) and its characterization in BMDMs and mice (25). Using the DKO as the base, we created TKO-D (*∆fbpA-∆sapM-∆dosR*), TKO-Z (*∆fbpA-∆sapM-∆zmp1*), and QKO (*∆fbpA-∆sapM-∆dosR-∆zmp1*) as described above. To confirm the successful deletion of genes in these strains, we performed PCR using primers specific to the gene loci (Fig. 1) and compared the results with those of the wild-type Mtb H37Rv. In addition to the *dosR* and *zmp1* loci, the PCR was also used to amplify the *sapM* locus in these mutants as an internal control. The PCR results revealed lower size bands of 165 bp, 380, and 998 bp for *sapM*, *dosR*, and *zmp1* genes, as opposed to 998 bp, 960 bp, and 2,880 bp in wild-type Mtb H37Rv, consistent with the engineered gene deletions. The deletion of genes in DKO, TKO-D, TKO-Z, and QKO was also confirmed by Southern (data not shown) and genome sequencing (accession # provided at the end). These confirmed mutant strains were used for further characterization.

### Mtb mutant vaccine strains show lower intracellular survival in BMDMs

**Fig 2:**
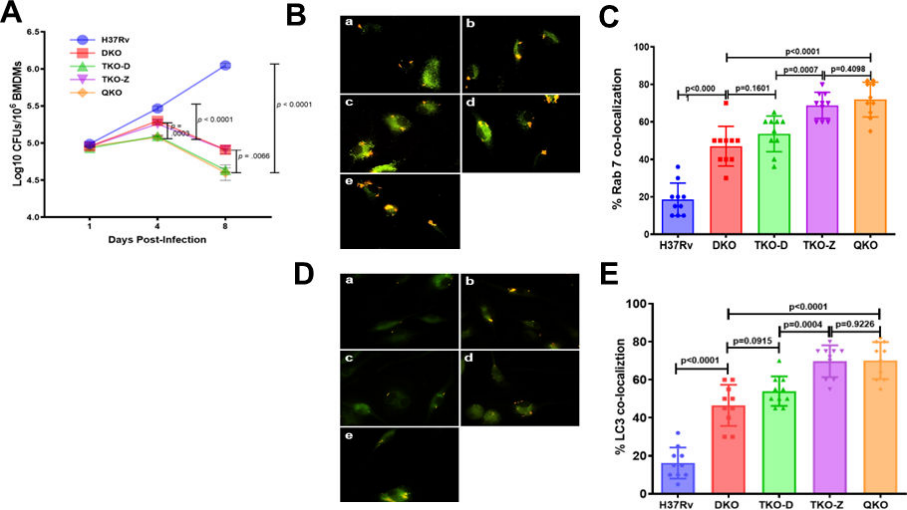
Rational deletion of genes reduces the intracellular survival and increases the processing of live vaccines through PL fusion and autophagy. (**A**) Deletion of genes in Mtb reduces intracellular viability/growth. BMDMs from naïve C57BL/6 mice were activated with IFN-γ and infected (MOI 1:1) with Mtb strains H37Rv, DKO, TKO-Z, TKO-D, or QKO and incubated. After 1, 4, and 8 days post-infection, cells were washed, lysed, and plated for viable colony counts (CFUs). Data represent the mean ± SD CFUs from triplicate. (**B**) Deletion of genes in Mtb leads to efficient processing through PL fusion. BMDMs from naïve C57BL/6 mice were infected with (a–e) *rfpH37Rv, rfpDKO, rfpTKO-D, rfpTKO-Z,* and *rfpQKO* (MOI = 1:1) for 4 h, washed, incubated for 24 h, and stained with primary antibodies to phagosomal maturation marker Rab7, followed by staining with FITC (green) conjugated secondary antibody. Red fluorescent Mtb colocalizing with Rab7 antibodies was scored using a Nikon TiE Fluorescence Microscope and Metaview Deconvolution Software. (**C**) Graph showing percent colocalization of Mtb with Rab7. Percent colocalization was determined by counting 50 macrophages per well, each with 1–3 mycobacteria, and averaging counts from triplicate chambers. One of three similar experiments is shown. (**D**) Deletion of genes in Mtb leads to efficient processing through autophagy. BMDMs from naïve C57BL/6 mice were infected with (a–e) *rfpH37Rv, rfpDKO, rfpTKO-D, rfpTKO-Z, and rfpQKO* (MOI = 1:1) for 4 h, washed, incubated for 24 h, and stained with primary antibodies to autophagy marker LC3, followed by staining with FITC (green) conjugated secondary antibody. Red fluorescent Mtb colocalizing with LC3 antibodies was scored using a Nikon TiE Fluorescence Microscope and Metaview Deconvolution Software. (**E**) The graph shows the percent colocalization of Mtb with LC3. Percent colocalization was determined by counting 50 macrophages per well, each with 1–3 mycobacteria, and averaging counts from triplicate chambers. One of three similar experiments is shown. Statistical significance was calculated using Student’s t-test. *P* values below 0.05 (*P* < 0.05) are considered significant.

The goal of the study was to develop attenuated vaccines from Mtb by rationally deleting genes. While Mtb survives and multiplies within the mononuclear phagocytes, attenuated Mtb strains have shown reduced ability to survive within macrophages (25, 37). Therefore, we determined the intracellular survival of the newly created Mtb mutant strains by infecting BMDMs and quantifying the bacterial burden at different time points of post-infection (1, 4, and 8 days). We used BMDMs infected with wild-type Mtb H37Rv and DKO as wild-type and attenuated controls. All mutant strains under study (TKO-Z, TKO-D, and QKO) showed a reduced bacterial load (CFUs) at 4 and 8 days of post-infection when compared to H37Rv (Fig. 2A). However, the survival rate of TKO-Z was no different from that of DKO, indicating that deletion of *zmp1* has no significant effect on the intracellular survival of Mtb. In contrast, a more pronounced reduction in bacterial load was observed in macrophages infected with TKO-D and QKO compared to those infected with TKO-Z and DKO, indicating that these two strains (TKO-D and QKO) are relatively more attenuated than the other strains. Furthermore, this also revealed that the deletion of the *dosR* gene has a significant impact on the intracellular survival of Mtb (Fig. 2A).

### Enhanced phagosomal maturation and autophagic response in BMDMs infected with Mtb mutant vaccine strains

We hypothesized that the relatively reduced intracellular survival of the Mtb vaccine strains is partly due to the processing of the strains by the BMDMs through the phagolysosomal and autophagic pathways. To test this, we performed phagosome maturation and autophagy processing of BMDMs infected with new vaccine strains (TKO-Z, TKO-D, and QKO) and controls (H37Rv and DKO) using Rab7 (a late endosomal marker) and LC3 (autophagosome marker), respectively. To enable the visualization of mycobacteria within macrophages, we expressed Red Fluorescent Protein (RFP) in all Mtb strains using a plasmid-borne *rfp* gene (41). BMDMs infected with RFP-expressing bacteria were colocalized with fluorescent Rab7 (green) and LC3 (green) markers at 48 h and 72 h post-infection. Robust colocalization (yellow) was noticed with both markers in cells infected with all the Mtb mutant strains (Fig. 2B and D). Quantitative analysis revealed that cells infected with the TKO-Z and QKO vaccine strains exhibited significantly higher colocalization of Mtb with Rab7 and LC3 compared to those infected with the wild-type H37Rv and other vaccine strains (DKO and TKO-D) (Fig. 2C and E). The TKO-D strain showed slightly elevated levels of colocalization with Rab7 and LC3 markers, but these levels were not significantly different from those in DKO-infected cells. Similarly, there was no significant difference between TKO-Z and QKO-infected cells in terms of colocalization with phagosomal and autophagy markers. Collectively, these results indicate that the enhanced colocalization of TKO-Z and QKO with Rab7 and LC3 markers compared to other strains was primarily due to the deletion of *zmp1* in these strains. The enhanced processing of the TKO-Z strain by macrophages, despite its relatively better intracellular survival, was surprising.

### Increased apoptotic cell death in BMDMs infected with Mtb vaccine strains

**Fig 3:**
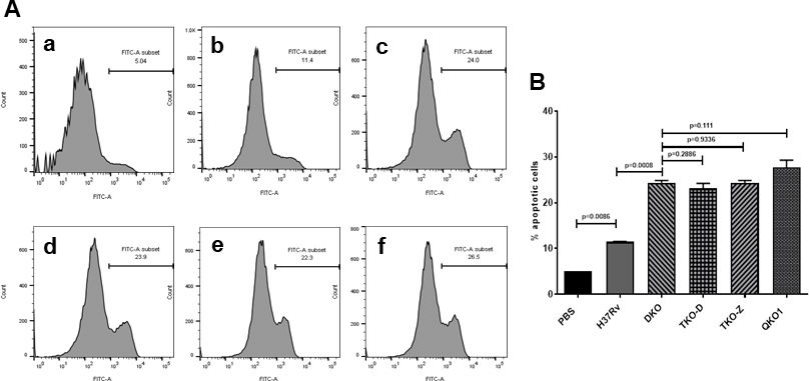
Rational deletion of genes in Mtb leads to a limited increase in apoptosis. (**A**) Flow cytometric analysis of FITC-Annexin V and propidium iodide (PI) staining. BMDMs (1 × 10^6^) from naïve C57BL/6 mice were infected with Mtb strains for 4 h, followed by washing and further incubation for 12–18 h. Cells were stained with FITC-Annexin V and PI and analyzed by flow cytometry. (a) Uninfected control, (b) H37Rv, (c) DKO, (d) TKO-D, (e) TKO-Z, and (f) QKO. Increased Annexin V positivity was observed in vaccine strains compared to H37Rv, indicating higher levels of apoptosis. (**B**) Quantification of total apoptotic cells (Annexin V positive) is represented as a bar graph. All four vaccine strains (DKO, TKO-D, TKO-Z, and QKO) induced significantly higher levels of apoptosis compared to wild-type H37Rv. Data represent mean ± SD from three independent experiments. Statistical significance was calculated using Student’s t-test. *P* values below 0.05 (*P* < 0.05) were considered significant.

To determine whether BMDMs infected with Mtb vaccine strains undergo apoptosis (programmed cell death), we analyzed the apoptosis of these infected cells using Annexin V staining followed by flow cytometry (40). Annexin V binds to phosphatidylserine, which is exposed on the outer leaflet of the plasma membrane during early apoptosis. The flow cytometry analysis (Fig. 3A) of the infected cells demonstrated that cells infected with vaccine strains (DKO, TKO-Z, TKO-D, and QKO) underwent significant apoptosis in comparison to cells infected with H37Rv. However, unlike phagosome maturation, there was no considerable difference among them (Fig. 3B), indicating that neither the deletion of *dosR* nor *zmp1* in Mtb has any synergistic effect on the induction of apoptosis in host cells.

### Enhanced antigen presentation by BMDMs infected with Mtb vaccine strains

**Fig 4:**
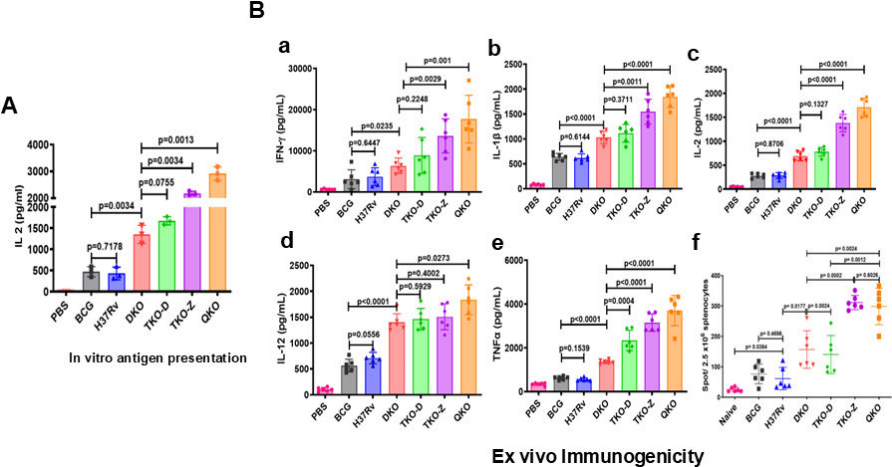
Rational deletion of genes in Mtb increases the immunogenicity of vaccine strains. (**A**) *In vitro* antigen presentation. BMDMs from naïve C57BL/6 mice were infected with BCG and Mtb strains (H37Rv, DKO, TKO-D, TKO-Z, and QKO) (MOI = 1:5) for 4 h and cocultured with BB7 T-cell hybridoma specific for Ag85B_241-256_ peptide. After 16 h, culture fluids were collected and assayed for IL-2 levels released by the BB7 cells in response to Ag85B peptide. (**B**) *Ex vivo* immunogenicity to vaccine strains. C57BL/6 mice were vaccinated with BCG, DKO, TKO-D, TKO-Z, and QKO vaccine strains (1 × 10^6^ subcutaneously) and the control H37Rv. After 30 days of post-immunization, mice were euthanized, and spleens were isolated. Splenocytes (2.5 × 10^5^/well) were plated and stimulated *in vitro* for 48 h with Mtb H37Rv whole-cell lysate (20 µg/mL). Supernatants from cultures were collected, and IFN-γ, (a) IL-1β, (b) IL-2, (c) IL-12 (d), and TNF (e) levels were determined by ELISA. ELISpot analysis for IFN-γ-producing splenocytes in vaccinated mice (f). C57BL/6 mice were vaccinated with BCG, DKO, TKO-D, TKO-Z, and QKO vaccine strains and control H37Rv (1 × 10^6^ subcutaneously). After 30 days post-immunization, mice were euthanized, and spleens were isolated. Splenocytes (2.5 × 10^5^/well) were plated and stimulated with a combination of Ag85B and CFP-10 peptides *in vitro* for 48 h. Ag85B/CFP-10 responsive IFN-γ-producing spleen cells were spotted using IFN-γ ELISpot plates following the manufacturer’s protocols. Statistical significance was calculated using Student’s t-test. *P* values below 0.05 (*P* < 0.05) were considered significant.

Since the Mtb mutant vaccine strains TKO-Z- and QKO-infected macrophages showed increased colocalization with Rab7 and LC3 markers, we predicted that cells infected with these strains would present Mtb antigens to T cells more effectively than cells infected with other mutants. To assess this, we performed an *in vitro* antigen presentation assay based on an epitope of the Ag85B antigen described before (12, 39). In this assay, BMDMs were independently infected with Mtb mutant vaccine strains, BCG, or wild-type H37Rv and cocultured with the BB7 CD4 T-cell line. The levels of antigen presentation were determined by measuring IL-2 levels in the culture supernatants of each infected cell culture. The data presented in Fig. 4A reveal that all culture supernatants from cells infected with the Mtb mutant strains had significantly higher levels of IL-2 compared to the supernatants of cells infected with the wild-type H37Rv or BCG, indicating that the macrophages process the mutant strains more effectively. However, as observed with phagosome maturation and autophagy markers, the macrophages infected with mutants differ in their presentation of antigens. While TKO-D-infected macrophages showed no significant difference from DKO-infected cells, macrophages infected with TKO-Z and QKO exhibited significantly higher levels of antigen presentation, as evident by the levels of IL-2. These results suggest that there exists an association between enhanced phagosomal processing and antigen presentation.

### Enhanced *ex vivo* immunogenicity by Mtb vaccine strains

We have previously reported that enhanced antigen presentation by macrophages infected with the DKO strain showed an increase in *in vivo* immunogenicity (25, 28). Therefore, we hypothesized that the TKO and QKO strains would also be more immunogenic in mice. C57BL/6 mice were immunized with the Mtb mutant vaccine strains (DKO, TKO-D, TKO-Z, and QKO) and controls (H37Rv and BCG), followed by *ex vivo* analysis of key pro-inflammatory and regulatory cytokines released by the splenocytes in response to Mtb whole-cell lysate as antigens. Significantly higher levels of IFN-γ, IL-1β, IL-2, IL-12, and TNF-α were found in the supernatants of splenocytes from mice immunized with Mtb mutant strains (Fig. 4Ba through e). ELISpot assay for IFN-γ also revealed similar results (Fig. 4Bf), indicating that the Mtb vaccine strains are highly immunogenic. Although the immunogenicity of the QKO strain seems to be superior to that of other strains, the data obtained were not statistically significant compared to the TKO-Z strain. Furthermore, the immunogenicity of TKO-D was not significantly different from the immunogenicity of DKO.

### Protective efficacy of Mtb vaccine strains in mice

**Fig 5:**
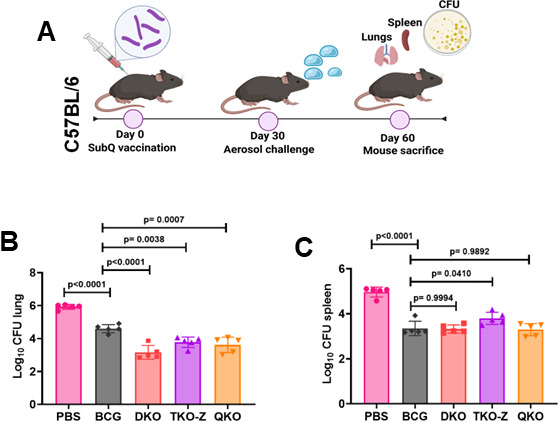
Rational deletion of genes in Mtb increases its vaccine efficacy over BCG. C57BL/6 mice were vaccinated with BCG, DKO, TKO-D, TKO-Z, and QKO vaccine strains (1 × 10^6^ subcutaneously). After 30 days post-immunization, mice were challenged with Mtb Erdman. Thirty days after post-challenge, mice were humanely euthanized, lungs and spleen were collected, and the bacterial counts (CFUs) were determined. (**A**) Schematic representation of experimental design. (**B and C**) Bar graphs show the bacterial load in the lungs and spleens of mice challenged with the Mtb Erdman. Statistical significance was calculated using one-way ANOVA with Dunnett’s post-test. *P* values below 0.05 (*P* < 0.05) were considered significant.

We recently reported that the DKO strain, which exhibits relatively higher immunogenicity than wild-type H37Rv, provides significant protection against Mtb infection in mice (28). To assess whether the TKO and QKO strains also show better protection, we immunized C57BL/6 mice with the Mtb vaccine strains (DKO, TKO-Z, or QKO) and control (BCG) and challenged them independently with the virulent Mtb Erdman strain. After 30 days post-challenge, the bacterial burden in the lungs and spleen was determined. The data presented in Fig. 5 reveal that the bacterial loads in the lungs of mice immunized with Mtb vaccine strains were significantly lower than those of mice immunized with BCG (Fig. 5A). However, the bacterial load in the spleen of mice immunized with Mtb vaccines is more or less similar to that of mice immunized with BCG (Fig. 5B). Notably, there was no significant difference in bacterial loads between mice immunized with Mtb vaccine strains, suggesting that the higher immunogenicity of TKO-Z and QKO had a minimal effect on protection against TB in mice.

### Survival of SCID mice after infection with Mtb vaccine strains

**Fig 6:**
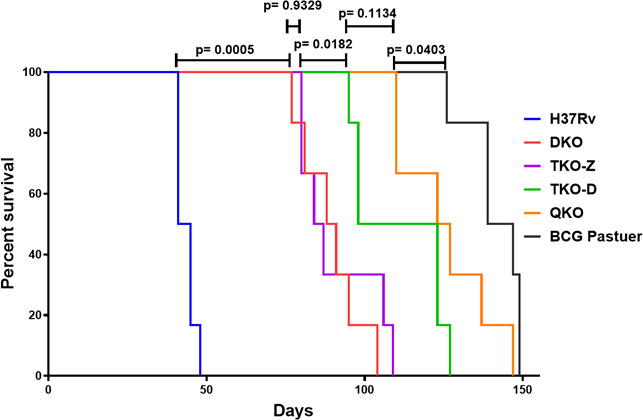
Rational deletion of genes in Mtb decreases its survival rate in SCID mice. SCID mice were infected with H37Rv, BCG, DKO, TKO-Z, TKO-D, and QKO through the intravenous route. Mice were monitored daily and euthanized upon reaching humane endpoints, including ≥20% wt loss, hunched posture, tremors, or ataxia. The figure shows the % survival of SCID mice. Statistical significance was determined using the log-rank (Mantel–Cox) test. *P* values below 0.05 (*P* < 0.05) were considered significant.

The safety of Mtb vaccines is important for their clinical application. Although the Mtb vaccine strains exhibited relatively lower intracellular survival in macrophages, suggesting attenuation, we assessed their relative virulence using SCID mice. These mice were infected intravenously with DKO, TKO-Z, TKO-D, and QKO, using wild-type Mtb H37Rv and BCG controls, and monitored for survival over time. They were euthanized upon reaching predefined humane endpoints, including ≥20% wt loss, hunched posture, tremors, or ataxia. The survival curves show that those infected with the wild-type H37Rv survived for the shortest time, around 43.5 ± 2.95 days post-infection. In contrast, mice infected with all vaccine strains, including BCG, survived longer than wild-type H37Rv (Fig. 6). Among the mutants, mice infected with DKO survived the least (89.33 ± 9.73 days), and mice infected with QKO survived the longest (125.67 ± 14.72 days), which is closer to the survival of BCG (141.5 ± 8.89). However, none of the SCID mice groups infected with Mtb mutant vaccine strains outperformed BCG in terms of survival.

## DISCUSSION AND CONCLUSION

Although several reasons have been attributed to the failure of the BCG vaccine against TB, it may partly be due to the lack of the region of deletion 1 (RD1), which encodes the major protein antigens ESAT-6 and CFP-10 (42, 43). Support for this notion comes from observations that subunit vaccines containing these proteins can mount a significant protection against TB (44–46), and BCG could be made more effective by expressing the RD1 region genes using recombinant methods (47, 48). Essentially, these data paved the way for the development of attenuated Mtb strains as vaccines against TB, a strategy that aims to strike a balance between safety and the retention of key antigenic determinants such as ESAT-6 and CFP-10, which are absent in BCG. Attenuation of Mtb is usually achieved through targeted deletions of virulence-related genes (49, 50). Indeed, we had previously constructed a novel DKO Mtb mutant by deleting two virulence-related genes, *fbpA* and *sapM*, which exhibited a significantly enhanced immune response and efficacy superior to that of BCG (25, 28). The current study focused on additional genetic modification of DKO to yield TKO and QKO strains by deleting *dosR* and *zmp1* virulence factors to create a vaccine with relatively enhanced immunogenicity and greater attenuation than DKO.

By deleting *zmp1* in the DKO, our objective was to improve the immunogenicity of DKO through increased PL fusion. Our results demonstrate that we have achieved this objective. Immunofluorescence colocalization in macrophages infected with TKO-Z and QKO strains, having deletions in *zmp1*, and stained with Rab7, exhibited strong PL fusion compared to DKO. Rab7 is a small GTPase that is pivotal for late‐endosomal/lysosomal trafficking and phagosome maturation, and an excellent marker for assessing phagosome maturation (11, 25, 51). Absence of significant colocalization of Rab7 with TKO-D strain reinforces that the effect is entirely due to the deletion of *zmp1* in TKO-Z and QKO. This is in line with the previous reports that the deletion of *zmp1* in Mtb and BCG increases phagosome maturation (52). Interestingly, however, our study shows that TKO-Z and QKO also colocalize with LC3, a biomarker associated with autophagosomes. We and others have used LC3 as a marker for assessing autophagy in Mtb-infected macrophages, which include the DKO strain (12, 28, 53). The increased autophagy in TKO-Z- and QKO-infected macrophages, but not in the TKO-D-infected macrophages, suggests that Zmp1 impedes autophagy in Mtb-infected macrophages, in addition to phagosome maturation. Although several Mtb proteins, such as Eis, PknG, PPE, PE_PGRS20, PE_PGRS47, and others have been implicated in the prevention of autophagy in Mtb-infected macrophages (54–56), a role for Zmp1 in autophagy is a novel observation. It may be that NLRP3-mediated inflammasome activation during the absence of Zmp1 has indirectly influenced autophagy in TKO-Z- and QKO-infected strains. Additional studies using H37Rv and the *∆zmp1* mutant strain in parallel may provide insights into the role of Zmp1 in autophagy.

Besides PL fusion and autophagy, the induction of apoptosis of macrophages by the vaccine is also considered essential to increase immunogenicity (57, 58). Mtb has multiple genes associated with the inhibition of apoptosis (59). Although the DKO showed significantly higher apoptosis compared to the wild-type Mtb H37Rv, the TKO-Z and QKO strains, despite enhanced phagosomal maturation and autophagy induction over the DKO, exhibit similar apoptosis induction to that of the DKO strain. This is not surprising because both Zmp1 and DosR have no known role in the inhibition of apoptosis. Additionally, there has been no report thus far that SapM phosphatase is associated with apoptosis. Therefore, we suggest that any apoptosis induction seen with the mutant vaccine strains may be due to the lack of expression of the FbpA protein in these strains. We previously observed the accumulation of reactive oxygen species (ROS) in macrophages infected with the *∆fbpA* strain (21), and it is possible that these ROS could have caused the apoptosis (60). In this context, we recognize that genetic complementation is critical for validating mutant phenotypes. However, this method poses significant challenges in our study because it uses Mtb mutants with multiple gene deletions. Consequently, we employed whole-genome sequencing to confirm the presence of the intended deletions in these mutants. The sequence data have been deposited in the NCBI database (SRR34500970–SRR34500968) and confirm that only the targeted gene deletions are present. Therefore, we conclude that the observed phenotypes are attributable to the specific gene deletions introduced. Nevertheless, earlier studies have complemented the single-gene Mtb mutants, like ∆sapM (27), ∆zmp1 (29), and ∆dosR (33, 61), to validate phenotypes such as PL fusion, inflammasome inhibition, and *in vivo* survival, which were also observed in this study.

Regardless, the deletion of *zmp1*, but not *dosR*, had a profound effect on the immunogenicity of Mtb mutants TKO-Z and QKO, which carried the *zmp1* deletion. The immunogenicity of a vaccine is primarily due to its presentation of antigens to effector cells, such as T cells, by APCs through the MHC-I and MHC-II molecules (16). On the other hand, the presentation of antigens through MHC-II heavily depends on the processing of the antigens by APCs through PL fusion or autophagy pathways (17). Consistent with enhanced PL fusion and autophagy, our *in vitro* and *ex vivo* results demonstrate a significant increase in the immunogenicity of TKO-Z and QKO strains. The *in vitro* immunogenicity is based on the presentation of the p25 epitope of Ag85B of Mtb (shared by BCG) to the BB7 CD4 T cells and subsequent release of IL-2 (62). This is a reliable marker for antigen presentation, and we have previously used this to determine the immunogenicity of BCG and Mtb strains, including the DKO strain (11, 12, 25). Remarkably, the *in vitro* immunogenicity was reflected by the *ex vivo* immunogenicity, and Mtb-specific antigens induced the release of key cytokines (IFN-γ, IL-1β, IL-2, IL-12, and TNF-α) in splenocytes from mice immunized with TKO-Z and QKO strains. These strong Th1-type cytokine responses may indicate the protective potential of TKO-Z and QKO vaccines, because vaccines with Th1 induction are central to eliminating the intracellular Mtb (6). Our recent transcriptomic study of BMDMs infected with the TKO-Z and QKO also showed a similar Th1 response (63). In addition, the transcriptomic data revealed the induction of IL-17 (Th17 response), another cytokine implicated in protection against TB (63). Altogether, deletion of *zmp1* makes the TKO-Z and QKO more immunogenic, which is similar to that reported for the BCG strain with deletion in *zmp1* (BCG∆*zmp1*) (52). This effect may be ascribed to the activation of the inflammasome in cells infected with TKO-Z and QKO, as Zmp1 suppresses inflammasome activation and delays phagosome acidification by degrading host phagosomal molecules (29).

What is fascinating may be that TKO-D, which struggles to survive in macrophages and is less virulent in SCID mice, elicits a weaker immune response than TKO-Z, which has the opposite traits. This contrast highlights the unique virulence strategies of DosR and Zmp1. DosR helps the bacteria persist by controlling approximately 50 genes under low-oxygen conditions (31, 32), while Zmp1 helps the pathogen evade immune detection by blocking inflammasome formation in antigen-presenting cells (29). These distinct mechanisms shape the immune responses observed with TKO-D and TKO-Z, showing that simply weakening a live bacterial vaccine does not guarantee greater immunogenicity. This finding reinforces the importance of carefully targeted gene deletions when designing Mtb-based vaccine candidates. We also rule out the idea that TKO-D’s limited immunogenicity is due to PhoP’s influence on the dosR gene, another two-component regulator (64–66). While PhoP does activate dosR and its hypoxia-related targets, only a handful of DosR-regulated proteins are antigens (67), and none are known to affect phagosomal maturation, inflammasome inhibition, or similar processes.

Disappointingly, however, our results indicate that the increased immunogenicity of the TKO-Z and QKO strains was not reflected in an increased protection against TB in mice, as both these strains appear to provide similar efficacy to that of DKO and TKO-D strains. This is not only surprising but also contradictory to a previous study that reported that the deletion of *zmp1* in BCG enhanced both immunogenicity and efficacy in mouse and guinea pig models (30). The reason why the increased immunogenicity of TKO-Z and QKO fails to lead to robust protection against TB in mice is unclear. One possibility is that, despite their higher immunogenicity, the quality of the immune response elicited by the TKO-Z and QKO strains, in terms of different effector T-cell types, may not be superior to that elicited by the DKO. More detailed studies with longer time points and with different animal models and OMICS immune profiles appear necessary to clarify the discrepancy in efficacy between DKO and TKO-Z/QKO strains.

Interestingly, however, survival studies on macrophages and SCID mice reveal that the rational deletion of genes is the most effective method for reducing the virulence of Mtb. In macrophages, the TKO-D and QKO strains show significantly lower survival rates compared to other strains. Reflecting this, the SCID mice infected with these strains show more prolonged survival than SCID mice infected with other strains, including DKO. The observation that the survival rate of SCID mice infected with QKO is closer to that of SCID mice infected with BCG may indicate that the QKO strain is a promising candidate for further modification. Previous studies have reported that the deletion of the *fadD26* gene provided significant attenuation for vaccines such as MTBVAC (68) and Mtb∆*sigE*-*fadD26* (69), which showed attenuation similar to or better than BCG in SCID mice studies. Our future studies will focus on the deletion of this gene in QKO to make it fully attenuated. The product *fadD26*, also *Rv2930*, is associated with the synthesis of cell surface lipids, phthiocerol dimycocerosates, and phenolic glycolipids, which are virulence factors (70).

Additionally, preclinical testing has been performed for numerous Mtb-derived vaccine candidates developed through single (e.g., Mtb∆*leuD*, Mtb∆*fbpA*, Mtb∆*sigE*, Mtb∆*lprG*), double (e.g., Mtb∆*panC*-*panD*, Mtb∆s*ecA2*-*lysA*, Mtb∆*sigE*-*fadD26*, Mtb∆*phoP*-*fadD2*6), or triple (e.g., Mtb∆*ptpA*-*ptpB*-*sapM*) gene deletions (reviewed in reference [71, 72]). One Mtb-derived vaccine, MTBVAC, based on the deletion of the *phoP* and *fadD26* genes, has advanced to a phase 2a clinical trial (73), and another vaccine candidate, the Mtb∆sigH vaccine, has been reported to reduce the bacterial burden by two log compared with BCG in challenge studies in Macaques (74). Although most candidates have been compared to the BCG vaccine in terms of efficacy, direct comparisons between them are lacking. This gap is partly attributable to the limited availability of published vaccine candidates for comparative studies due to proprietary restrictions. Nevertheless, such head-to-head comparisons are essential to identify the most promising candidates for clinical trials. We will address this in the future by comparing the QKO vaccine, following its refinement for attenuation, with vaccines such as MTBVAC, including the creation of an MTBVAC-equivalent strain in the laboratory.

Overall, our comprehensive evaluation demonstrates that the targeted deletion of key virulence regulators from Mtb yields live‐attenuated vaccine candidates that consistently outperform BCG across multiple immunological assays. This includes stronger Th1 cytokine responses *ex vivo,* greater CFU reductions in the lungs following aerosolized *M. tuberculosis* challenge, and increasing attenuation following multiple gene knockouts in SCID mice. Although all Mtb mutant strains showed better immune responses and lung protection than BCG, the QKO strain was highly attenuated, highlighting the potential of this targeted gene-deletion approach. Therefore, we propose that combining gene deletions that weaken Mtb’s ability to evade the immune system and survive within the host can generate vaccine candidates that are safer and offer longer-lasting protection against TB than BCG.

## Data Availability

Genomic sequence data for the Mtb mutants were submitted to the Sequence Read Archive (SRA/NCBI) database under BioProject number PRJNA1290405, accession numbers SRR34500970 and SRR34500968.

## AFTER EPUB

*[This article was published on 21 April 2026 with an error in the author byline. The author's name was corrected in the current version, posted on 23 April 2026.]*
